# Hepatocellular Carcinoma: From Molecular Basis to Novel Treatment Approaches

**DOI:** 10.1155/2019/4970731

**Published:** 2019-03-03

**Authors:** Hikmet Akkız

**Affiliations:** Professor of Gastroenterology and Hepatology, Head of Gastroenterology and Hepatology, Çukurova University, Turkey

Hepatocellular carcinoma (HCC) is among the most prevalent and lethal cancers in the world and is the fifth most common cancer and the second leading cause of cancer-related deaths. HCC incidence has increased dramatically during the last decade worldwide. HCC is endemic in East Asia and sub-Saharan Africa where the major risk factors are hepatitis B virus (HBV) and hepatitis C Virus (HCV) infection. Aflatoxin B1 is a relevant cofactor for HCC in sub-Saharan Africa. In the United States and Western Europe, Nonalcoholic Steatohepatitis is an emerging risk factor for the development of HCC.

Recently, Genome-Wide Association Study (GWAS) and Next Generation Sequencing (NGS) technologies have significantly improved our understanding of the molecular pathogenesis of HCC. Hepatocarcinogenesis is driven by interaction between host genetic polymorphisms, environmental factors including metabolic syndrome, aflatoxin B1, alcohol consumption, and viral factors including HBV and HCV infection. Key molecular drivers involved in hepatocarcinogenesis have been demonstrated. Molecular alterations at genomic, transcriptomic, and epigenomic level have been shown to be drivers in hepatocarcinogenesis. Genomic instability is key driver in hepatocarcinogenesis that may result in copy number alterations and somatic mutations. Epigenomic alterations causing DNA mutilation, histone modification, and chromatin remodeling regulate gene expression at the genome. Telomere and Telomerase have a key role in HCC development. Telomerase reverse transcriptase promoter (TERT) mutation is an early somatic alteration in hepatocarcinogenesis. Telomerase reactivation occurs in approximately 90% of HCC patients due to TERT promoter mutation, TERT amplification, and HBV insertion into the TERT promoter. Inactivation of p53 pathway, alterations in cell cycle signaling pathway, activation of Wnt/B-Catenin signaling and oxidative stress pathways, epigenetic alterations causing chromatin remodeling, and activation of Akt-mTOR-MAPK signaling have been shown to promote HCC progression.

Barcelona Clinic Liver Cancer (BCLC) staging system is the most used staging system in the world for estimating the prognosis of HCC patients and contributes to clinicians choice of an evidence-based allocation of curative and palliative treatments. According to BCLC staging system, potential curative treatments such as ablation, hepatic resection, and liver transplantation are standard options in stage 0 and A HCC patients. Although BCLC A stage is quite specific, BCLC stage B HCC (intermediate stage) is characterized by high heterogeneity which shows a significant challenge in terms of determining the most effective therapy. BCLC staging system recommends transarterial chemoembolization (TACE) for BCLC B patients. However, potential curative treatments may provide more favorable clinical outcomes in some BCLC B patients with preserved liver function.

The advanced stage HCC (BCLC C) includes an extremely heterogeneous patient population characterized by extrahepatic metastasis, macrovascular invasion, a wide range of Child-Pugh scores (A5-B9), and Eastern Cooperative Oncology Group performance status (PS 0-2) and systemic therapy with the multikinase inhibitor, sorafenib, being recommended in patients with advanced stage HCC.

This special issue includes relevant research articles and review articles. In the review article titled “Pleiotropic Effects of Heparins: From Clinical Applications to Molecular Mechanisms in Hepatocellular Carcinoma” published by P. Korhan et al., the authors summarize the state of knowledge whereby heparin may crosstalk with molecules playing a role in hepatocarcinogenesis, and highlight new experimental and clinical research-related personalized therapy in patients with cancer at risk of thromboembolism.

The research article published by G. Odabas et al. showed that Plexin C1 distinguishes HCC cells of epithelial characteristics from those with the mesenchymal phenotype and HCC tissue overexpresses Plexin C1 compared to stroma.

In the review article titled “Molecular Pathogenesis of Nonalcoholic Steatohepatitis- (NASH-) Related Hepatocellular Carcinoma” written by O. Kutlu et al., the investigators provide comprehensive knowledge of NASH-related hepatocarcinogenesis and highlight molecular signaling pathways that may have a role in HCC development.

H. Akkiz et al. have demonstrated the association between portal vein thrombosis and maximum tumor diameter, multifocality, and AFP in the large cohort study.

In the review article on local ablation therapies for HCC, S. Shiina et al. have given updated knowledge about various ablation techniques and compared them to surgical modalities.

